# Development and Functional Characterization of Recombinant Mussel Adhesive Protein for Anti-Oxidative and Anti-Aging Therapeutic Applications

**DOI:** 10.3390/ijms262411947

**Published:** 2025-12-11

**Authors:** Suhan Wi, Seon-A Lim, Jin-Yeong Jung, Hyungmo Yang, Sun-Ae Lee, Kyounghun Choi, Ju-Ryeong Kim, Moo-Hak Lim, Yong-Hyun Kim, Jaehong Park, SeongMin Ha, Yun Heo

**Affiliations:** Nature Gluetech Co., Ltd., 196, Gasan Digital 1-ro, Geumcheon-gu, Seoul 08502, Republic of Korea; wisu@natureglue.com (S.W.); plok016@natureglue.com (S.-A.L.); ginyung92@natureglue.com (J.-Y.J.); ttkkyang91@natureglue.com (H.Y.); sun@natureglue.com (S.-A.L.); kyonghun11@natureglue.com (K.C.); joo@natureglue.com (J.-R.K.); moo@natureglue.com (M.-H.L.); yongkim22@natureglue.com (Y.-H.K.); jaehong@natureglue.com (J.P.); hsng20@natureglue.com (S.H.)

**Keywords:** mussel adhesive proteins, antioxidant activity, anti-aging, oxidative stress, ROS, stability, skin permeability, 3D culture, recombinant protein, *E. coli*

## Abstract

Reactive oxygen species (ROS) are well-known as major contributors to skin aging, and the application of antioxidants to suppress ROS has been increasingly emphasized in the field of cosmetics. Traditionally, plant-derived or synthetic antioxidants have been predominantly used. More recently, antioxidant, anti-aging, and anti-inflammatory effects of mussel extracts or mussel-derived hydrolysates have also been explored and proposed for cosmetic applications. However, there is still a lack of scientific research on the antioxidant and anti-aging effects of specific mussel foot proteins, particularly engineered recombinant proteins such as NGT-M001 (hybrid fp-151). Natural mussel adhesive proteins are characterized by an amino acid composition rich in tyrosine and catechol groups such as DOPA, which are believed to possess potent antioxidant activity. In this study, we quantitatively evaluated the antioxidant and anti-aging properties of a recombinant mussel adhesive protein (rMAP) lacking DOPA modification, and a rationally designed protein, NGT-M001. Antioxidant activity was assessed using ABTS and DPPH radical scavenging assays, while anti-aging potential was evaluated through collagenase and hyaluronidase inhibition assays. In the ABTS assay, the antioxidant capacity per molar unit of NGT-M001 was 27.76-fold higher than that of Trolox. NGT-M002 (pvFP-5), NGT-M003 (pvFP-5 fragment) and NGT-M004 (FP-1 fragment) exhibited 9.08-fold, 2.84-fold, and 8.54-fold higher activities, respectively. In the DPPH assay, NGT-M001 and NGT-M004 were not detected, whereas NGT-M002 and NGT-M003 showed 3.45-fold and 1.59-fold higher activity than Trolox, respectively. These findings suggest that recombinant mussel adhesive proteins such as NGT-M001 exhibit antioxidant activity and may serve as promising next-generation bioactive ingredients for functional medicine and cosmetic applications to improve skin health and radical scavenging.

## 1. Introduction

In recent years, the fields of cosmetics, pharmaceuticals, and food science have actively pursued the discovery of innovative bioactive compounds—both plant-derived and synthetic—that can enhance skin health and mitigate signs of aging [1]. Oxidative stress induced by reactive oxygen species (ROS) has been unequivocally identified as a principal factor contributing to skin aging, inflammation, and a range of dermatological conditions [2]. ROS are highly reactive molecules produced during normal cellular metabolic processes as well as in response to external stimuli such as ultraviolet (UV) radiation and environmental pollutants [3,4,5]. These reactive species cause oxidative damage to skin cells, thereby accelerating the aging process [6,7,8]. Additionally, the excessive accumulation of ROS has been strongly associated with carcinogenesis, thereby positioning antioxidants as a critical area of investigation within the fields of nutrition, pharmaceuticals, and dermatology [9,10,11,12,13].

Common antioxidants employed in these contexts include L-ascorbic acid, glutathione, N-acetyl cysteine, tocopherol, retinol, and resveratrol [14,15,16]. Nevertheless, these compounds are characterized by inherent limitations such as poor solubility, chemical instability, and notably low permeability through the skin barrier [17,18,19,20,21]. To address these challenges, research efforts have expanded to explore plant-derived extracts, chemical modifications, and protein-based antioxidants, including superoxide dismutase (SOD) and various reductases [22,23]. Despite these advances, fundamental obstacles related to scalable production and efficient penetration into the epidermis or cellular compartments persist, thereby constraining their practical application in cosmetic and therapeutic formulations [24].

In this context, mussel adhesive proteins (MAPs) have emerged as promising candidates for the development of novel antioxidants and anti-aging agents. Extracted from the byssal threads of mussels, MAPs comprise a diverse group of proteins, including fp-1 through fp-6 [25]. Among these, fp-6 has been reported to exhibit significant antioxidant activity in DPPH radical scavenging assays, attributed to its high content of cysteine and tyrosine residues [26,27]. Moreover, proteins such as fp-1, fp-3, and fp-5, which are enriched in 3,4-dihydroxyphenylalanine (DOPA), have demonstrated strong adhesive properties alongside potential antioxidant effects, although quantitative evaluations remain limited [28,29,30,31]. Furthermore, comprehensive investigations into proteins like fp-5 derived from *Perna viridis*, which contain both cysteine residues and GFP-like domains, are currently lacking, particularly with respect to their antioxidant and anti-aging functionalities [32,33].

To date, research on MAPs has predominantly focused on their adhesive properties as biomaterials, with limited systematic evaluation of their antioxidant capacity, anti-aging effects, and stability as cosmetic ingredients. Recombinant MAPs such as fp-1 and fp-5, particularly fp-151 produced via *Escherichia coli* expression systems, offer industrial scalability [34,35,36,37,38,39].

However, this has been a limitation for use of the recombinant proteins in cosmetics and pharmaceuticals, because there are no DOPA residues to induce the adhesion [40,41]. DOPA exerts antioxidant activity by participating in electron transfer reactions with tyrosine, thereby helping to prevent excessive oxidation and maintain protein integrity [42,43]. Furthermore, the marine-origin materials market is expected to increase with a CAGR of 7.13% per year up to dollars (USD) 10.59 billion by 2030 (and beyond) [44]. This rapid market growth highlights the increasing attention being paid to marine-derived biomaterials, particularly MAPs, due to their unique properties and potential for novel applications, thereby underscoring the need for further development of MAPs.

Accordingly, this study was conducted to systematically evaluate the antioxidant and anti-aging activity of recombinant MAPs generated from *E. coli* expression systems. We also evaluated quantitatively their stability and skin penetration as a cosmetic ingredient. Through revealing the special molecular entities of marine MAPs and their antioxidative actions, we suggest that recombinant MAPs may have distinct advantage than common antioxidants. Mussel-derived proteins, despite lacking DOPA residues, are proposed as functional ingredients capable of effectively mitigating ROS-induced damage.

## 2. Results

### 2.1. Expression and Purification of Recombinant Protein NGT-M001, NGT-M002, NGT-M003 and NGT-M004

**Figure 1:**
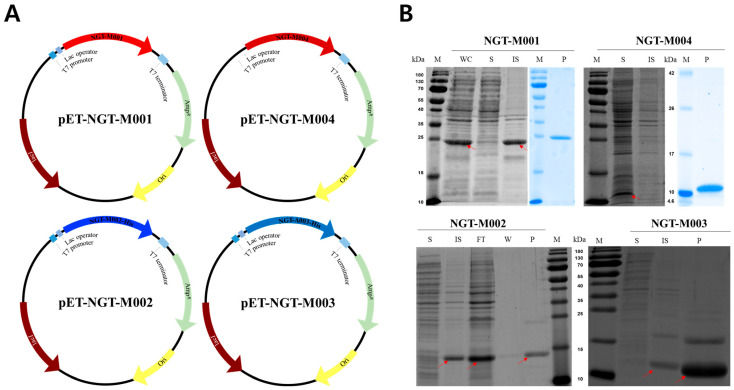
Recombinant mussel adhesive protein expression and purification in *E. coli* BL21 (DE3). (**A**) *E. coli* expression vector construction. (**B**) Expression and purification of recombinant MAPs. The red arrow indicates the band position of each recombinant MAPs. Lanes: M, marker; WC, whole cell lysate; S, soluble fraction; IS, insoluble fraction; W, wash fraction; P, purified protein.

The expression profiles of four recombinant polypeptides (NGT-M001, NGT-M002, NGT-M003, and NGT-M004) were analyzed by Coomassie Brilliant Blue staining, as shown in Figure 1. When expressed in *Escherichia coli* BL21 (DE3) under induction with 1 mM IPTG at 37 °C (Figure 1A), NGT-M001 (~22.6 kDa), NGT-M002 (~15 kDa), and NGT-M003 (~8 kDa) were predominantly present in the insoluble fraction (IS), whereas NGT-M004 (~8 kDa) was primarily detected in the soluble fraction (S). The observed molecular weights were consistent with theoretical values predicted by ProtParam (http://web.expasy.org/protparam/, cited on 25 September 2025), supporting the feasibility of recombinant expression in *E. coli*, as previously reported [38]. NGT-M001 and NGT-M004 are classified as intrinsically disordered proteins (IDPs) and may retain their bioactivity even when expressed in the IS fraction, depending on downstream processing [45,46]. In contrast, NGT-M002 and its derivative NGT-M003, both originating from Perna viridis, contain three and one EGF-like domains, respectively, and possess 18 and 10 cysteine residues, suggesting that proper folding is essential for their functionality [47,48]. When expressed in the IS fraction, these proteins are more likely to misfold and lose their activity. Given that strategies like N-terminal tagging (e.g., MBP, SUMO, or NEXT), host strain engineering, and low-temperature expression are commonly used to improve solubility, we intend to incorporate these methods in future research [49,50,51,52]. In the present study, *E. coli* BL21 (DE3) cells were utilized, and protein expression was induced using 0.1 mM IPTG at 37 °C. As shown in Figure 1B, this condition successfully facilitated insoluble expression of the target proteins. NGT-M001, NGT-M002 and NGT-M003 insoluble expressed in *E. coli* BL21 (DE3) required solubilization with high concentrations of guanidine-HCl, urea, and reducing agents for refolding. In the future, the expression pattern can be optimized according to the intended application, enhancing the practical utility of recombinant mussel adhesive proteins.

### 2.2. Quantitative Evaluation of Antioxidant Activity

**Figure 2:**
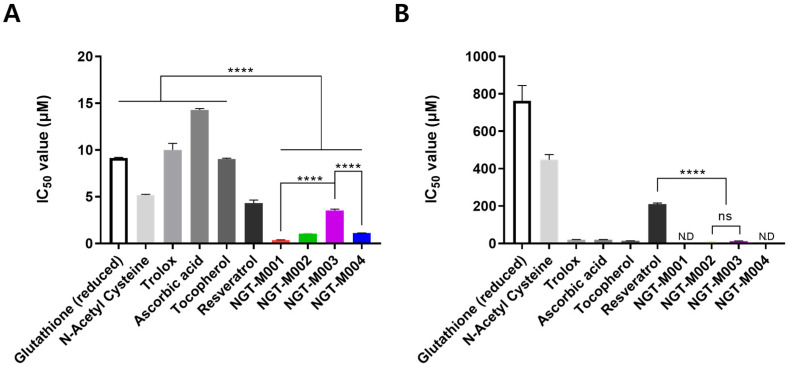
Comparison of antioxidant activities in ABTS (**A**) and DPPH assay (**B**). The data are expressed as the mean ± standard deviation (SD, n = 3), and difference evaluations are shown as **** *p* < 0.0001.

Antioxidant activity was assessed under hydrophilic conditions using the ABTS^•+^ radical scavenging assay, and under hydrophobic conditions using the DPPH assay. Recombinant mussel adhesive proteins (NGT-M001 to NGT-M004) and tested antioxidant cosmetic ingredients were evaluated based on their IC_50_ values, which represent the concentration required to reduce radical activity by 50%. Lower IC_50_ values indicate stronger antioxidant capacity. In the ABTS assay, the IC_50_ values of NGT-M001, NGT-M002, NGT-M003, and NGT-M004 were 0.38 ± 0.05, 1.06 ± 0.00, 3.52 ± 0.15, and 1.10 ± 0.01 μM, respectively. In contrast, the DPPH assay revealed activity only for NGT-M002 and NGT-M003, with IC_50_ values of 5.66 ± 1.12 and 12.48 ± 1.29 μM, respectively (Figure 2). The DPPH assay of compounds NGT-M001 and NGT-M004 was conducted up to 5 mM, but no IC_50_ values were observed. To benchmark antioxidant capacity, Trolox was used as a reference standard, and Trolox Equivalent Antioxidant Capacity (TEAC) was calculated. In the ABTS assay, resveratrol showed the highest activity among tested antioxidants (2.32 μM TE/μM), whereas NGT-M001 to NGT-M004 exhibited TEAC values of 26.41, 9.43, 2.84, and 9.05 μM TE/μM, respectively. In the DPPH assay, tocopherol showed the highest activity among tested antioxidants (1.37 μM TE/μM), while NGT-M002 and NGT-M003 recorded TEAC values of 3.43 and 1.559 μM TE/μM, respectively (Table 1). Furthermore, among the antioxidant-associated amino acids L-Cysteine, L-Tyrosine, and L-DOPA, the DPPH assay demonstrated that L-Tyrosine, tested at concentrations up to 2.5 mM, exhibited no detectable activity. Consequently, it is inferred that the antioxidant properties observed in NGT-M001 and NGT-M004 are attributable to the presence of Tyrosine, which does not interact with DPPH radicals. As a result, under hydrophilic conditions, NGT-M001 exhibits approximately 11.34-fold higher antioxidant capacity than resveratrol, and under hydrophobic conditions, NGT-M002 shows 2.49-fold greater activity than tocopherol, highlighting their potential as promising bioactive ingredients for cosmetic applications.

### 2.3. ABTS Radical Scavenging Kinetics Curves and Persistence Effect

**Figure 3:**
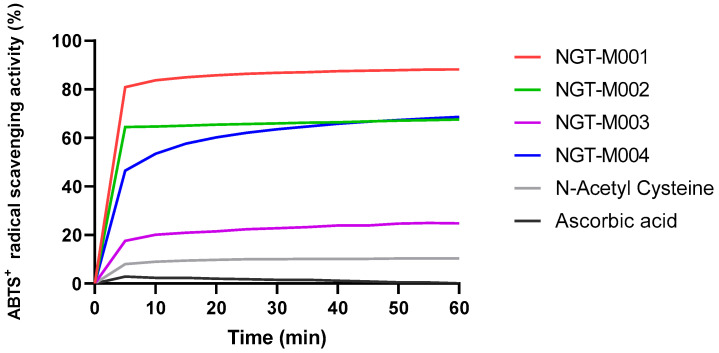
ABTS^+^ radical scavenging kinetics curves of MAPs.

To confirm the antioxidant durability of recombinants against evaluated antioxidants, ABTS^•+^ radical scavenging activity was examined at a 1 μM constant concentration. N-acetyl cysteine kept about 10% scavenging up to 60 min, whereas L-ascorbic acid did not maintain even 3% after 5 min, instead showing a rapid decrease. This quick inactivity loss is believed to be due to the structural instability of ascorbic acid. However, NGT-M001, NGT-M002, NGT-M003, and NGT-M004 showed stronger radical scavenging activity at the equivalent concentration, and the enhancement with prolonging occurred in a continuous manner (Figure 3). Taken together, the above results provide clear evidence for the higher initial and sustained antioxidant activity of rMAPs over evaluated antioxidants, especially L-ascorbic acid. From these results, V_0_ (initial reaction rates) and AUC (cumulative antioxidant persistence) were also calculated (Table 2). The initial rates of L-ascorbic acid and N-acetyl cysteine were 0.24 and 0.39%/min, respectively, whereas NGT-M001 to NGT-M004 had corresponding efficiencies of 8.37, 6.47, 2.00, and 5.35%/min. Antioxidant efficiency compared to L-ascorbic acid (AEAC) for NGT-M001 to NGT-M004 was 35.06-, 27.1-, 8.41-, and 22.4-fold higher, respectively. The AUC values for antioxidant longevity were 88%·min for L-ascorbic acid and 564.47%·min for N-acetyl cysteine. Under the same conditions, NGT-M001 to NGT-M004 exhibited substantially higher AUC values of 4953.7, 3795.13, 1293.4, and 3552.18%·min, respectively. Compared to L-ascorbic acid, these represent approximately 56.29-, 43.12-, 14.69-, and 40.36-fold increases in cumulative antioxidant activity (Figure 3). In sum, these results confirm that recombinant proteins, especially NGT-M001, possess higher initial antioxidant activity and more durable free radical scavenging ability than classical antioxidants.

### 2.4. Anti-Aging Effects of rMAPs

**Figure 4:**
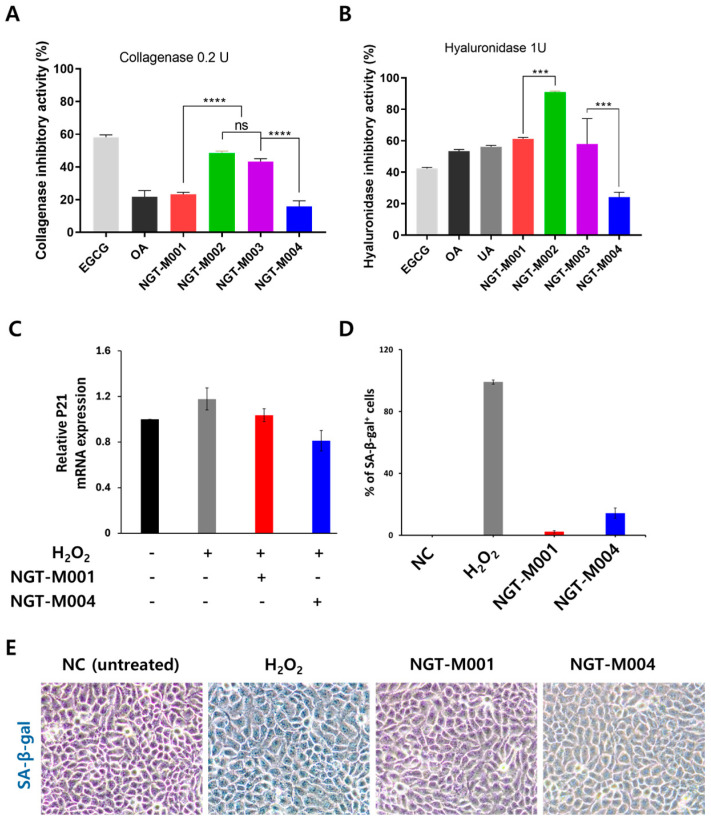
In vitro anti-aging activity test with a sample concentration of 500 μM. Collagenase inhibitory activity (**A**) and Hyaluronidase inhibitory activity (**B**) of rMAPs. HaCaT cells were treated with 50 μM H_2_O_2_ for 2 h to induce P21 expression, followed by treatment with MGT-M001 (1.1 μM, 25 μg/mL), NGT-M004 (3.125 μM, 25 μg/mL), and the relative P21 mRNA expression was compared by qRT-PCR (**C**). Manual quantification of SA-β-gal-positive cells (**D**) and representative micrographs of SA-β-gal-stained cells (**E**). The data are expressed as the mean ± standard deviation (SD, n = 3), and difference evaluations are shown as *** *p* < 0.001, **** *p* < 0.0001. ns indicates not significant.

In this study, the anti-aging potential of recombinant MAPs was evaluated by assessing their inhibitory activities against collagenase and hyaluronidase. Based on prior research, positive controls including epigallocatechin gallate (EGCG), oleanolic acid (OA), and Ursolic acid (UA) were shown to inhibit collagenase and hyaluronidase activities in a dose-dependent manner. For comparative clarity, enzyme inhibition was assessed at a fixed concentration of 500 μM (Figure 4 and Figure S1). Collagenase inhibition was measured using 0.2 U/mL collagenase, with EGCG and OA showing inhibition rates of 58.22 ± 1.36% and 21.72 ± 3.82%, respectively. Recombinant proteins NGT-M001, NGT-M002, NGT-M003, and NGT-M004 exhibited inhibition rates of 23.32 ± 1.1%, 48.44 ± 1.28%, 43.26 ± 1.18%, and 15.79 ± 3.53%, respectively. These results indicate that MAPs possess 40%, 83%, 74%, and 27% of the collagenase inhibitory activity of 500 μM EGCG, respectively (Figure 4A). Hyaluronidase inhibition was assessed using 1 U/mL hyaluronidase. EGCG, OA, and UA inhibited the enzyme by 42.37 ± 0.7%, 2.37 ± 1.08%, and 56.18 ± 0.87%, respectively. In comparison, NGT-M001, NGT-M002, NGT-M003, and NGT-M004 showed inhibition rates of 61.31 ± 0.83%, 90.99 ± 0.61%, 57.91 ± 16.18%, and 24.24 ± 2.92%, respectively. Except for NGT-M004, all recombinant proteins demonstrated significantly higher hyaluronidase inhibition than the positive control UA. Specifically, the MAPs retained 109.13%, 161.95%, 103.07%, and 43.15% of the hyaluronidase inhibitory activity of 500 μM UA, respectively (Figure 4B). HaCaT cells were treated with 50 μM H_2_O_2_ for 2 h to induce P21 expression, followed by treatment with 25 μg/mL rMAPs, and the relative P21 mRNA expression was compared. As a result, P21 expression in H_2_O_2_-treated HaCaT cells was approximately 1.2-fold higher than that of the untreated control. NGT-M001 showed a level comparable to NT, whereas NGT-M004 suppressed P21 expression down to 0.8-fold. However, NGT-M002 and NGT-M003 exhibited aggregation under biological conditions, indicating that they were not suitable for cell-based experiments (Figure 4C). Under identical conditions, analysis of SA-β-gal activity showed that nearly all HaCaT cells exposed to H_2_O_2_ (≈99%) were SA-β-gal-positive. In contrast, only 2.3 ± 0.8% of NGT-M001 cells and 14.3 ± 3.3% of NGT-M004 cells were positive. Notably, unlike the P21 mRNA expression results shown in Figure 4C, the SA-β-gal activity analysis indicated that NGT-M001 displayed stronger anti-senescent activity than NGT-M004 (Figure 4D,E). Accordingly, the rMAPs presented here are not only designed to be inhibitors of ECM-degrading enzymes but they also dramatically decrease the levels of p21 in cells and exert an anti-senescence effect on cells. Taken together, these combined effects indicate that rMAPs can be potential anti-aging biomaterials focusing on both ECM integrity and senescence-related pathways.

### 2.5. Antioxidant Stability Analysis of rMAPs

**Figure 5:**
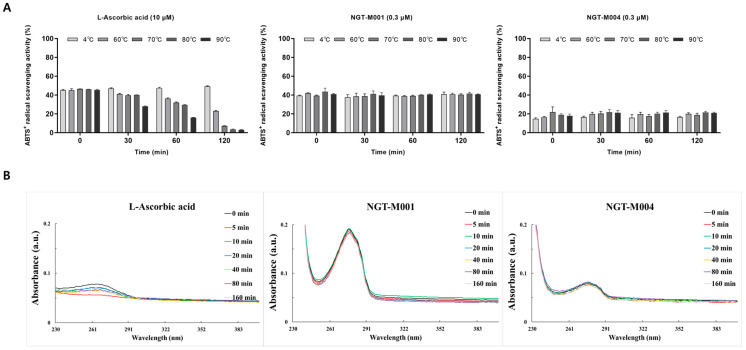
Antioxidant stability analysis of NGT-M001 and NGT-M004. (**A**) Thermostability evaluation of L-ascorbic acid, NGT-M001, and NGT-M004 in DW solution at various temperatures (4, 60, 70, 80 and 90 °C) using the ABTS assay. (**B**) Photostability evaluation of L-ascorbic acid, NGT-M001, and NGT-M004 in DW solution at different time points after UVB exposure (225 μW/cm^2^), followed by UV-Vis absorption spectrum measurement.

During the preliminary assessment, NGT-M002 and NGT-M003 demonstrated significant precipitation following heat treatment and dissolution in phosphate-buffered saline (PBS), thereby limiting their applicability for further investigation. Consequently, subsequent analyses were concentrated on NGT-M001 and NGT-M004. To evaluate thermostability, solutions containing 10 μM L-ascorbic acid and markedly lower concentrations (0.3 μM, approximately 33-fold less) of NGT-M001 and NGT-M004 were prepared in distilled water and subjected to thermal exposure at 4, 60, 70, 80, and 90 °C for a duration of 120 min. Antioxidant activity was subsequently quantified using the ABTS radical scavenging assay. For photostability evaluation, samples were irradiated with UVB light at an intensity of 225 μW/cm^2^, followed by absorbance measurements across the 230–400 nm wavelength spectrum. The findings indicated that both NGT-M001 and NGT-M004 preserved stable radical scavenging activity throughout the entire temperature range tested (4–90 °C) over the 120-min incubation, demonstrating exceptional thermostability. In contrast, L-ascorbic acid, despite being employed at a concentration 33 times higher (10 μM), exhibited a rapid decline in antioxidant efficacy with increasing temperature and incubation time, with activity reduced by approximately 50% after 120 min at 60 °C (Figure 5A). Furthermore, SDS-PAGE analysis of NGT-M001 and NGT-M004 samples subjected to 120 min of heat treatment revealed consistent band patterns and intensities across all temperature conditions, confirming their molecular weights of 22.6 kDa and 8 kDa, respectively (Figure S2). These results suggest that both proteins maintain their structural integrity under thermal stress and are thus amenable to stable application in cosmetic manufacturing processes. In standard cosmetic formulation protocols, heating at 60–70 °C for 1–2 h during dispersion and mixing frequently results in thermal degradation of evaluated antioxidants such as L-ascorbic acid, leading to approximately 50% loss of activity. Conversely, NGT-M001 and NGT-M004 retained full antioxidant functionality under identical conditions, indicating high thermal stability and suitability for incorporation into heat-intensive manufacturing environments. Photostability assessments revealed that L-ascorbic acid exhibited immediate instability upon UV exposure, with absorbance decreasing by over 90% after 5 min (67.5 mJ/cm^2^) of irradiation. In contrast, NGT-M001 and NGT-M004 maintained highly stable absorbance profiles despite prolonged UV exposure (Figure 5B). Although evaluated antioxidants like L-ascorbic acid possess potent radical scavenging capabilities, their limited photostability—as corroborated in this study—often results in premature degradation via photolytic mechanisms prior to effective neutralization of reactive oxygen species (ROS). The enhanced photostability observed in the rMAPs is likely attributable to their proteinaceous molecular structures, which confer greater resistance to UV-induced damage relative to small-molecule antioxidants.

### 2.6. In Vitro Cell Cytotoxicity and Ex Vivo Skin Permeability Evaluation of the MAPs

**Figure 6:**
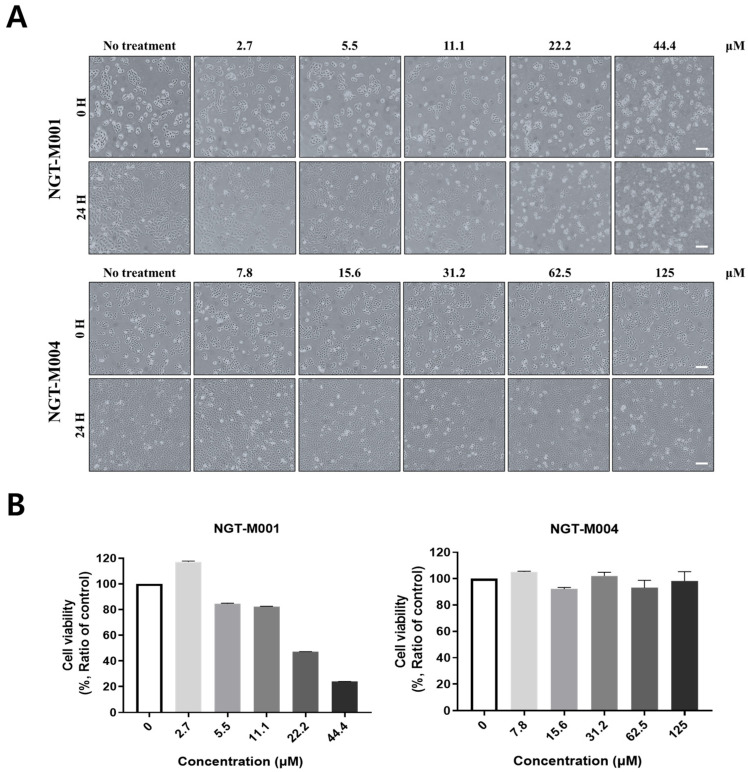
In vitro cell cytotoxicity test of NGT-M001 and NGT-M004. (**A**,**B**) Cell morphology, scale bar = 100 μm and MTT cell viability evaluation of NGT-M001 and NGT-M004 at various concentrations. The data are expressed as the mean ± standard deviation (SD, n = 3).

Cytotoxicity was assessed by HaCaT cells treated with different concentrations of NGT-M001 and NGT-M004 observed under an inverted microscope. Compared to the controls, a significant dose-dependent decrease in cell viability was observed (Figure 6). Skin permeation studies were performed using human cadaver skin mounted on a FDC system. Meanwhile, the normal morphology of HaCaT cells exposed to NGT-M001 was confluent with the control up to 11.1 μM; however, at higher concentrations (>11.1 μM), intracellular granulation and cell death were noticed. Comparatively, NGT-M004-treated cells displayed no morphology and density changes when compared with the controls at all concentrations tested except for granule formation (Figure 6A). MTT assay results also backed up these findings. NGT-M001 maintained approximately 80% cell survival at 11.1 µM, and demonstrated over 100% cell viability (up to approximately 120%) even at the lowest tested concentration (2.7 μM), suggesting that NGT-M001 may have a stimulatory effect on keratinocyte proliferation and viability at low concentrations. In contrast, no cytotoxicity was detected for NGT-M004 even at the highest tested concentration of 125 μM (Figure 6B). The observed cytotoxicity differences between NGT-M001 and NGT-M004 are likely attributable to their distinct net charge and hydrophobicity profiles. Since NGT-M004 is a domain of NGT-M001, less affinity towards the cell membrane might explain its decreased cytotoxicity. The Franz diffusion cell (FDC) study was performed using cadaver skin inside the donor chamber with the receiver chamber containing PBS solution. L-ascorbic acid, gelatin, NGT-M001 and NGT-M004 were placed onto the surface of cadaver skin in the donor chamber and incubated at 37 °C for 16 h. The quantity of target protein which had penetrated the receptor compartment was determined. As expected, NGT-M001 showed better skin permeability than NGT-M004 as permeation percents of 1.88 ± 0.16 and 1.05 ± 0.1 (Table 3). In addition, the skin penetration efficiency of NGT-M001 was approximately 2.6-fold higher than that of 60 kDa gelatin, while showing about 0.5-fold penetration compared to L-ascorbic acid. It is notable that, although NGT-M001 was 2.8-fold higher in molecular dimension than NGT-M004, the former showed approximately 1.8-fold more skin permeability facilitated compared to the latter. This increased penetrability is believed to result from its large positive charge, which might help the penetration into skin.

### 2.7. Enhanced Biocompatibility of NGT-M001 and HA Coacervate in 3D Cell Culture

**Figure 7:**
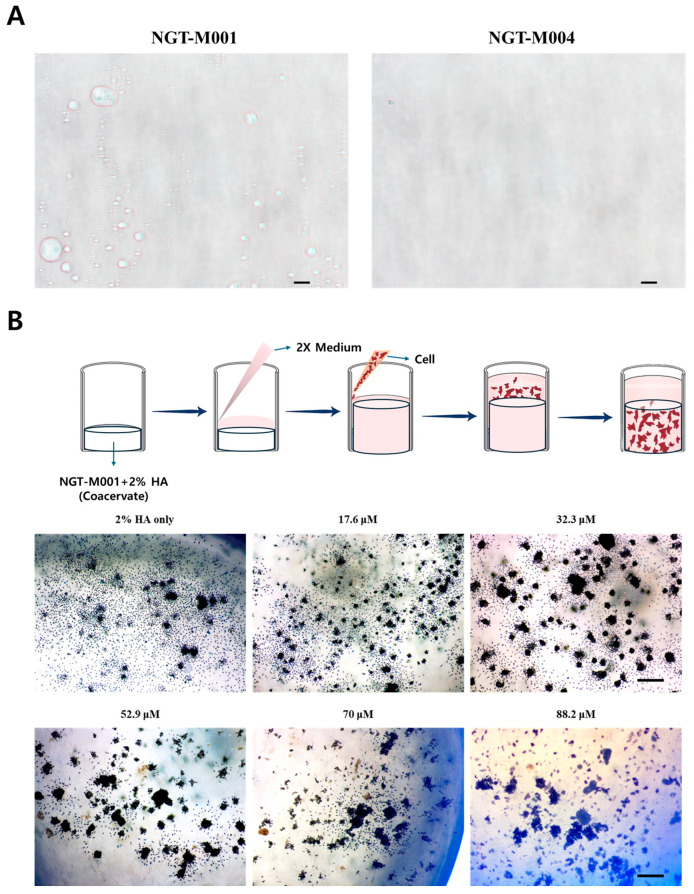
NGT-M001 coacervate promotes cell attachment. NGT-M001 and NGT-M004 (each final 1 mg/mL) coacervate formation with 1% Hyaluronic acid, scale bar = 100 μm (**A**). Schematic of shown the coacervate 3D culture system and microscopic observation after MTT staining of L929 cell 3D cultures at various concentrations of NGT-M001, which exhibits excellent coacervate forming ability, scale bar = 300 μm (**B**).

There are relatively higher contents of tyrosine and lysine in NGT-M001 in comparison to NGT-M004, which may explain the differences in cytotoxicity between them. To address whether the lysine, which interacts with HA (a major component of human skin), affects fibroblast behavior in the coacervate, we first assessed the coacervation potential of NGT-M001 and NGT-M004 (Figure 7A). When each protein was mixed with 1% HA at a 1:1 ratio and observed with an optical microscope, NGT-M001 showed coacervation to produce turbid and small liquid droplets. In contrast, NGT-M004 was unaltered. As shown in Figure 7B, NGT-M001 was mixed with 2% HA and added into a 48-well plate, then cultured with L929 cells for 72 h. After the addition of MTT reagent and microscopic examination, cell density was significantly increased compared to the control group treated only with 2% HA solution. Notably, at NGT-M001 concentrations ranging from 32.3 to 52.9 μM exceeding the previously determined CC50 value of 23.9 μM for HaCaT cells—the cell condition was markedly improved. The effect was most apparent at 52.9 μM where cells were visibly attached to the coacervate scaffold (Figure 7B).

## 3. Discussion

This study investigated the potential application of rMAPs devoid of DOPA as active ingredients in pharmaceutical and cosmetic formulations. We evaluated their antioxidant and skin anti-aging properties, stability, and skin permeability, alongside cytotoxicity assessments utilizing an *Escherichia coli* expression system. The findings demonstrated that these proteins exhibited markedly enhanced antioxidant and anti-aging activities, coupled with increased stability. Furthermore, their transdermal penetration was significantly greater compared to evaluated antioxidants.

The antioxidant effects observed in this study revealed distinct antioxidant characteristics, dividing into two groups: NGT-M001/NGT-M004 and NGT-M002/NGT-M003. NGT-M001 and its derivative protein NGT-M004 have high tyrosine content but lack cysteine, showing activity in the ABTS radical assay but low activity in the DPPH radical assay (Figure 2). The IC_50_ values derived from the ABTS and DPPH assays exhibited considerable variation, which can be ascribed to the differing chemical characteristics and reaction mechanisms inherent to the two radical species. Specifically, ABTS demonstrates greater solubility in aqueous media and interacts rapidly with both hydrophilic and lipophilic antioxidants, whereas DPPH exhibits reduced reactivity in aqueous environments, resulting in elevated IC_50_ values. Although it is well-documented that ABTS and DPPH assays can produce substantially divergent IC_50_ values in certain instances, our investigation revealed that standard antioxidants, including ascorbic acid and Trolox, yielded comparable IC_50_ values across both assays. This observation suggests that the observed discrepancies are dependent on the specific compounds tested rather than representing a universal phenomenon. This is explained by the vulnerability of the phenolic hydroxyl group structure of tyrosine to oxidants [53], which accounts for the antioxidant effect in ABTS^+^. In contrast, NGT-M002 and NGT-M003 contain both tyrosine and cysteine, exhibiting strong antioxidant activity against both ABTS and DPPH radicals. The sulfur atom in cysteine contributed to an effective radical scavenging mechanism [54,55]. Additionally, it was discussed that the ABTS assay can evaluate activity in both hydrophilic and lipophilic compounds, whereas the DPPH assay is limited to measuring only lipophilic compounds [56,57]. Through this structure–activity relationship analysis, it was found that the antioxidant activity of recombinant proteins is closely related to their amino acid composition. In particular, the hydrophilic properties of NGT-M001 and NGT-M004 limit their interaction with DPPH, while overall, they show strong and sustained antioxidant activity, suggesting high potential as functional antioxidant materials.

The anti-aging potential of recombinant MAPs was demonstrated through their inhibitory activities against collagenase and hyaluronidase (Figure 4). Collagenase is a major mediator of skin aging through collagen degradation, and its active site includes histidine residues such as HIS218 and HIS228, as well as hydrophobic residues like LEU181. The high collagenase inhibitory activity observed in NGT-M002 and NGT-M003 is presumed to be related to the structural characteristics of their EGF-like domains [58,59]. Notably, although the concentration of hyaluronidase was approximately fivefold higher than that of collagenase, MAPs showed greater inhibitory effects against hyaluronidase. This is attributed to both direct enzyme inhibition and the structural protection of the substrate, hyaluronic acid, thereby enhancing its stability. The Mechanism of Hyaluronidase Inhibition by rMAPs, the exceptionally high hyaluronidase inhibition by NGT-M002 (90.99%) compared to the positive control ursolic acid (56.18%), warrants deeper mechanistic investigation. Unlike small-molecule inhibitors that operate primarily through direct active-site binding, rMAPs employ at least two complementary mechanisms: First, the direct enzyme inhibition pathway involves covalent and non-covalent interactions between NGT-M002’s surface residues and the hyaluronidase catalytic center. The 18 cysteine residues in NGT-M002 are particularly significant, as their thiol groups can form disulfide bonds or hydrogen bonds with histidine and other nucleophilic residues in the enzyme’s active site. This not only blocks substrate access but also may alter the enzyme’s conformational state, reducing catalytic efficiency. Additionally, the EGF-like domains provide extended interaction surfaces via hydrophobic patches and electrostatic interactions with the enzyme’s surface. The relative contributions of each mechanism can be evaluated through a series of planned experimental approaches, including surface plasmon resonance (SPR) binding kinetics, molecular docking analyses, and site-directed mutagenesis. These methodologies will enable precise identification of the protein–protein interaction interfaces and allow quantification of the effects of individual amino acid residues on inhibitory efficacy. Furthermore, preceding these experimental investigations, we performed protein–protein interaction simulations utilizing artificial intelligence and deep learning techniques. Computational analyses suggested that NGT-M002 and NGT-M003 could selectively bind to specific sites on hyaluronidase through LightDock (Figure S3) [60,61,62,63]. Second, the substrate protection mechanism involves the formation of protein–polysaccharide complexes. Our coacervation studies (Figure 7) demonstrate that NGT-M001 (with high lysine content) forms stable complexes with hyaluronic acid through electrostatic interactions. By analogy, NGT-M002’s multiple cysteines and charged residues likely form similar protective complexes with HA substrate, rendering the polysaccharide inaccessible to enzymatic degradation even if direct enzyme inhibition is incomplete. We also found that among the rMAPs we manufactured, NGT-M001 and NGT-M004 not only inhibit ECM-degrading enzymes but also show anti-aging effects in H_2_O_2_-induced senescent cells by suppressing P21 expression (Figure 4C). The challenge model used was HaCaT cells, which carry an intrinsic p53 mutation, yet they still showed clear differences compared with the untreated NC group. Under these experimental conditions, we observed a significant change in p21 mRNA expression. Furthermore, SA-β-gal analysis suggested that NGT-M001 and NGT-M004 help protect cells by reducing oxidative stress caused by H_2_O_2_ (Figure 4D,E).

NGT-M001-type MAPs, which include many lysine residues, demonstrated excellent water retention in hyaluronic acid and showed specificity for coacervate formation (Figure 7A). On the other hand, NGT-M004, which did not form a coacervate, is thought to be a fragment of NGT-M001 with inadequate size and positive charge. The coacervate structure of NGT-M001 is presumed to promote skin moisture, and together with collagen, might be expected to prevent wrinkles [64,65,66,67]. Additional experiments to investigate this effect are scheduled. Moreover, intradermally formed coacervates display antioxidant and enzyme-inhibitory activities and are likely to retain moisture for a prolonged period and act as a crosslinker between cells and collagen fibers in the extracellular matrix (ECM), thereby contributing to long-lasting skin elasticity [68]. Collagenase and hyaluronidase are enzymes known to degrade skin ECM components, including collagen and hyaluronic acid, both of which are essential for maintaining skin elasticity, moisture retention, barrier function against external stimuli, and tissue repair [69,70,71]. Due to their high lysine content, these proteins are well known as biocompatible molecules since they can be degraded by endogenous proteases such as trypsin and chymotrypsin. Given the relatively high transdermal penetration efficiency observed, it should be considered that systemic circulation of MAPs may potentially elicit immune sensitization responses. Although lysine-rich MAPs are known to be biocompatible, further human safety and efficacy evaluations are required to assess the risk of immunogenicity upon systemic exposure. These results demonstrate that MAPs can act as inhibitors of these degrading enzymes, supporting their potential use for skin hydration and elasticity in various regions. Thus, MAPs can be a useful material for both pharmaceuticals and antioxidants/anti-aging functional cosmetics.

In this study, stability assays (Figure 5), two pvFP-5 proteins with good solubility (NGT-M001 and NGT-M004) were chosen for further characterization, while the others (NGT-M002 and NGT-M003) displayed strong aggregation characteristics under biological environment. This choice was based on previous studies indicating that pvFP-5 proteins may cause aggregation or instability in certain conditions such as 5% acetic acid [72,73,74]. These observations indicate that future strategies aimed at improving solubility will be essential for fully realizing the biological activity and physicochemical potential of this protein family. NGT-M001 and NGT-M004 exhibited high thermal stability attributable to their structural features, which are associated with increased hydrogen bonding, salt bridges, disulfide bonds, and hydrophobic interactions. In particular, their intrinsically disordered structures confer resistance to heat-induced conformational changes, thereby providing relative thermal and photostability compared with the commonly used L-ascorbic acid (Figure 5). These characteristics enhance the potential utility of these proteins in diverse cosmetic formulations and may help bridge the gap between the functional efficacy of active ingredients and the benefits perceived by consumers. Additionally, when incorporated into sunscreen products, NGT-M001 and NGT-M004 are anticipated to provide stable improvements in UV protection; targeted experimental validation of this effect will be undertaken in future research.

Practical concentration ranges were proposed for pharmaceutical applications, with consideration of cytotoxicity, and for cosmetic use, based on skin permeability (Figure 6). NGT-M001 demonstrated greater cytotoxicity than NGT-M004 upon direct cellular exposure; however, its toxicity diminished in relation to skin penetration, consistent with findings from a three-dimensional culture model employing coacervates formed by combining hyaluronic acid (HA) and NGT-M001 (Figure 7B). The cytotoxicity associated with NGT-M001 is hypothesized to result from electrostatic disruption of the cell membrane due to its pronounced positive charge, whereas co-formulation with HA mitigates this effect by neutralizing the charge, thereby reducing cytotoxicity. Furthermore, compared to HaCaT cells, L929 cells exhibited heightened sensitivity to cytotoxic effects but also served as supportive substrates that facilitated stable cell attachment and proliferation, consequently diminishing the apparent cytotoxic response. These observations suggest that, following skin penetration, residual hyaluronic acid may interact with NGT-M001 to form structures analogous to skin boosters or dermal fillers, which exhibit antioxidant properties while concurrently enhancing hydration and wrinkle amelioration. This rationale informed the application of three-dimensional culture models in the present investigation.

We recognize that cost-effectiveness constitutes a critical determinant for commercial adoption, alongside bioactivity. rMAPs are categorized as high-performance proteins characterized by inherently complex production processes, which pose challenges in maintaining price parity with conventional antioxidants. Nonetheless, we are actively implementing multiple strategies to improve their economic feasibility. These include scale-up optimization through the transition from laboratory-scale to industrial-scale production to realize economies of scale; process enhancement via advanced bioprocessing technologies aimed at increasing yield and reducing manufacturing costs; and improvements in manufacturing efficiency by streamlining purification and formulation procedures to minimize overall expenses. It is important to highlight that the bioactivity of rMAPs may compensate for their higher unit cost by enabling lower concentrations in final product formulations. Moreover, as production technologies advance and market penetration expands, the cost differential is anticipated to diminish substantially, mirroring trends observed with other bioengineered proteins within the cosmetics industry.

In summary, mussel-derived adhesive proteins, even in the absence of DOPA residues, are capable of reducing both intracellular and extracellular reactive oxygen species via tyrosine residues, demonstrate anti-aging activity, and substantially overcome the stability and skin permeability limitations associated with tested antioxidants. These attributes position them as promising candidates for development as environmentally friendly therapeutic agents and as primary or adjunctive components in cosmetic formulations.

## 4. Materials and Methods

### 4.1. Synthesis of Recombinant Mussel Adhesive Protein

The recombinant mussel adhesive proteins used in this study were produced by Nature Gluetech Co., Ltd., Seoul, Republic of Korea. Briefly, *E. coli* DH5α (#CP011, Enzynomics, Daejeon, Republic of Korea) was used for cloning and plasmid preparation, while *E. coli* BL21 (DE3) (#CP111, Enzynomics, Daejeon, Republic of Korea) was used for expression of recombinant proteins. Plasmids were constructed by polymerase chain reaction (PCR) amplification and subcloning target genes into expression vectors. The recombinant strains were cultured, and protein expression was induced with Isopropyl-β-D-thiogalactopyranoside (IPTG; #EI05931, Biosynth Carbosynth, Compton, UK). Proteins were purified from cell lysates using affinity and ion exchange chromatography under standard conditions.

#### 4.1.1. *E. coli* Strains and General Culture Conditions

The strains, plasmids, and oligonucleotide primers used in this study are listed in Table 4. *E. coli* DH5α was used for DNA experiments, recombinant protein expression was performed in *E. coli* BL21 (DE3). Terrific Broth (TB; #MB-T1556, Kisanbio, Seoul, Republic of Korea) medium supplemented with antibiotics (100 μg/mL ampicillin (#MB-A4128, Kisanbio, Seoul, Republic of Korea) or 50 μg/mL kanamycin (#MB-K4390, Kisanbio, Seoul, Republic of Korea)) was used for *E. coli* culture at 37 °C with shaking at 200 rpm in an incubator (Jeiotech, Daejeon, Republic of Korea).

#### 4.1.2. Plasmid Construction

The pET-NGT-M001 hybrid fp-151 plasmid was provided by Prof. H.J. Cha (POSTECH). The NGT-M004 gene, encoding a six-tandem-repeat fp-1 decapeptide, was PCR-amplified using the primers (forward: 5′-GGGCATATGGCGAAACCGAGCTATCCG-3′; reverse: 5′-GGGCTCGAGTTTGTATGTCGGCGGGTAAGACG-3′) from the pET-NGT-M001 plasmid as a template and subcloned into pET-22b (+) (#69744, Novagen, Madison, WI, USA) using NdeI (#1161A, Takara, Shiga, Japan) and XhoI (#1094A, Takara, Shiga, Japan) restriction sites, resulting in pET-NGT-M004. NGT-M002 (pvFP-5) gene (GenBank accession number: AGZ84276) was codon optimized for *E. coli*, chemically synthesized (Macrogen, Seoul, Republic of Korea) and subcloned into pET-22b (+) using NdeI and XhoI restriction sites with 6X histidine, resulting in pET-NGT-M002. Other plasmids containing genes were constructed based on PCR.

#### 4.1.3. Expression and Purification

Recombinant *E. coli* BL21 (DE3) strains transformed with the constructed plasmids were incubated in TB media supplemented with 100 μg/mL ampicillin at 37 °C in a shaking incubator. The expression of recombinant protein was induced at 0.6−0.8 OD_600nm_ by adding 1 mM IPTG. The cells were further cultivated at 37 °C for 12 h, centrifuged at 4 °C and 4000g for 10 min, and resuspended in 50 mM sodium phosphate buffer supplemented with 300 mM NaCl (#000S0484, Samchun, Seoul, Republic of Korea) and 10 mM imidazole (#I5513, Sigma-Aldrich, St. Louis, MO, USA) (pH 8.0). Cell lysates were prepared by disrupting the cell suspensions with an ultrasonic dismembrator (VCX500, Sonics and Materials, Newtown, CT, USA) for 20 min at 20% amplitude on ice after. For fractionation, the lysates were centrifuged at 4 °C and 10,000g for 10 min. The supernatant was designated the soluble fraction (S) while the pellet was designated the insoluble fraction (IS). The soluble fraction was mixed with Ni^2+^-nitrilotriacetic acid agarose beads (Ni-NTA; #30230, Qiagen, Hilden, Germany), insoluble expression proteins were solubilized for room temperature overnight incubation in 6 M Urea, 10 mM DTT, 50 mM tris buffer (pH 8.0). The supernatant harvested after centrifugation at 10,000× *g*, 4 °C for 10 min, filtered using 0.45 μm filter, and loaded onto a 40 mL XK16/40 column (#28988938, Cytiva, Marlborough, MA, USA), packed with SP Sepharose FF resin (#17072901, Cytiva, Marlborough, MA, USA), equilibrated in the same buffer as the protein sample. The solubilized protein was purified at room temperature using a linear NaCl gradient (0–1000 mM) under denaturing and reducing conditions over 20 column volumes. The eluted protein was subsequently subjected to refolding through a series of extensive dialysis steps conducted at 4 °C. Initially, dialysis was performed in a buffer containing 10 mM Tris (pH 8.0), 2 M urea, and 250 mM NaCl, followed by dialysis in the identical buffer formulation devoid of urea. Ultimately, the refolded protein underwent dialysis in 2% acetic acid prior to lyophilization. And the NGT-M001 was purified according to the manufacturer’s instructions. Protein purity was evaluated using 15% (*w*/*v*) SDS-PAGE followed by Coomassie Brilliant Blue staining, yielding a purity level exceeding 90% by Bio-Rad image lab 6.0 software. The protein concentration was determined spectrophotometrically at 280 nm, employing an extinction coefficient (ϵ) of NGT-M001 (65,560 M^−1^ cm^−1^), NGT-M002 (36,885 M^−1^ cm^−1^), NGT-M003 (19,995 M^−1^ cm^−1^) and NGT-M004 (17,880 M^−1^ cm^−1^).

#### 4.1.4. Protein Gel Electrophoretic Analysis

Sample proteins were separated by sodium dodecyl sulfate−polyacrylamide gel electrophoresis (SDS-PAGE). The proteins on the gel were visualized by staining with 0.1% Coomassie Brilliant Blue R-250 (#1610400, Bio-Rad, Hercules, CA, USA) in 10% acetic acid, 50% methanol, and 40% distilled water, followed by destaining with 10% acetic acid, 50% methanol, and 40% distilled water.

### 4.2. Radical Scavenging Activity ABTS Assay and Kinetics Analysis

The ABTS radical cation (ABTS^•+^) solution was prepared by mixing 7.4 mM 2,2′-azino-bis(3-ethylbenzothiazoline-6-sulfonic acid) diammonium salt (ABTS; #194430, Sigma-Aldrich, St. Louis, MO, USA) with 2.6 mM potassium persulfate (#379824, Sigma-Aldrich, St. Louis, MO, USA) and allowing the mixture to stand in the dark at room temperature for 12–16 h. Before use, the ABTS^•+^ solution was diluted with phosphate-buffered saline pH 7.4 (PBS; #SH30256.01, Cytiva, Marlborough, MA, USA) to an absorbance of 0.70 ± 0.03 at 734 nm. For the assay, 50 μL of each sample was mixed with 950 μL of the diluted ABTS^•+^ solution and incubated in the dark at 25 °C for 10 min [75]. Each sample, NGT-M001 to NGT-M004 and the following antioxidants: L-Ascorbic acid (#95210, Sigma-Aldrich, St. Louis, MO, USA), N-Acetyl-L-Cysteine (NAC; #A7250, Sigma-Aldrich, St. Louis, MO, USA), L-Glutathione reduced (GSH; #G6013, Sigma-Aldrich, St. Louis, MO, USA), 6-Hydroxy-2,5,7,8-tetramethylchroman-2-carboxylic acid (Trolox; #H0726, TCI, Tokyo, Japan), DL- α-Tocopherol (#T0251, TCI, Tokyo, Japan), Resveratrol (#R0071, TCI, Tokyo, Japan), all-trans-Retinol (#347071000, Thermo Fisher Scientific, Waltham, MA, USA). The absorbance was measured at 734 nm using a UV–visible spectrophotometer (Genesys10S, Thermo Fisher Scientific, Waltham, MA, USA). The ABTS radical scavenging activity (%) was calculated using the following equation:

$$\mathrm{Scavenging}\;\mathrm{activity}\;(\%)=\left(1-\frac{A_{\mathrm{sample}}}{A_{\mathrm{control}}}\right)\times 100$$

The Trolox equivalent antioxidant capacity (TEAC) was expressed as µmol Trolox Equivalent (TE)/uM of rMAPs or compounds.

Kinetics analysis was 1 uM test sample for time course in ABTS assay using kinetics method of UV-Vis spectrophotometer (Synergy Neo2 Multi-Mode Reader, BioTek, Winooski, VT, USA). The kinetics V_0_ (%/min) was calculated using the following equation:

$$V_{0}=\left.\frac{dy(t)}{dt}\right.$$

Then kinetic area under the curve (AUC, %·min) was calculated using Trapezoidal rule the following equation:

$$AUC=\sum_{i=0}^{n-1}\frac{y_{i}+y_{i+1}}{2}\left(t_{i+1}-t_{i}\right)$$

### 4.3. Radical Scavenging Activity DPPH Assay

The DPPH radical solution was prepared by dissolving 0.1 mM 2,2-diphenyl-1-picrylhydrazyl (DPPH; #044150.03, Thermo Fisher Scientific, Waltham, MA, USA) in 100% methanol. Prior to use, the solution was adjusted to an absorbance of 0.98 ± 0.03 at 517 nm. For the assay, 50 μL of each sample was mixed with 150 μL of the DPPH solution and incubated at 25 °C for 30 min in the dark. After incubation, the absorbance was measured at 517 nm using a UV–visible spectrophotometer [76,77]. The DPPH radical scavenging activity (%) was calculated using the following equation:

$$\mathrm{Scavenging}\;\mathrm{activity}\;(\%)=\left(1-\frac{A_{\mathrm{sample}}}{A_{\mathrm{control}}}\right)\times 100$$

### 4.4. Collagenase Inhibition Assay

To evaluate collagenase inhibitory activity was measured following method [78,79,80]. 10 μL of collagenase from *Clostridium histolyticum* (2 U/mL; #C8051, Sigma-Aldrich, St. Louis, MO, USA), 60 μL of Tricine buffer (composition in mM: 50 Tricine (#T0682, TCI, Tokyo, Japan), 10 CaCl_2_ (#C5080, Sigma-Aldrich, St. Louis, MO, USA), 400 NaCl; pH 7.5), and 10 μL of each test sample (0–1 mM) were mixed. Then, 20 μL of the substrate solution, 1 mM N-[3-(2-furyl)acryloyl]-Leu-Gly-Pro-Ala (#F5135, Sigma-Aldrich, St. Louis, MO, USA) in Tricine buffer was added, and the mixture was incubated at 37 °C for 20 min. After incubation, the absorbance was measured at 335 nm using a UV–visible spectrophotometer. The following inhibitors were used as positive controls in the enzyme inhibition assay: Epigallocatechin gallate (EGCG; #PHR1333, Sigma-Aldrich, St. Louis, MO, USA), Oleanolic acid (OA; #O05504, Sigma-Aldrich, St. Louis, MO, USA), Ursolic acid (UA; #U6753, Sigma-Aldrich, St. Louis, MO, USA)

The collagenase inhibition activity (%) was calculated using the following equation:

$$\mathrm{Collagenase}\;\mathrm{inhibitory}\;\mathrm{activity}\;(\%)=\left(1-\frac{{Abs}_{\mathrm{sample}}}{{Abs}_{\mathrm{control}}\;}\right)\times 100$$

A standard curve was generated, and the IC_50_ value was determined accordingly.

### 4.5. Hyaluronidase Inhibition Assay

To evaluate hyaluronidase inhibitory activity, the following reagents were prepared: test samples (0–500 μM); hyaluronidase from bovine testes (50 U/mL; #H3506, Sigma-Aldrich, St. Louis, MO, USA) dissolved in 20 mM phosphate buffer (pH 7.0) containing 77 mM NaCl and 0.01% bovine serum albumin (BSA; #R3961, Promega, Madison, WI, USA); 0.03% hyaluronic acid (HA, 720 kDa; #1.4, Hyundai Bio-land, Cheongju, Republic of Korea) solution containing 300 mM phosphate buffer (pH 5.35); and stop buffer containing 24 mM sodium acetate (#241245, Sigma-Aldrich, St. Louis, MO, USA), 79 mM acetic acid (#A6283, Sigma-Aldrich, St. Louis, MO, USA), and 0.1% BSA. Briefly, 25 μL of each test sample was mixed with 3 μL of hyaluronidase solution and incubated at 37 °C for 10 min. Then, 12 μL of 300 mM phosphate buffer (pH 5.35) was added, followed by a second incubation at 37 °C for 10 min. Next, 10 μL of the 0.03% hyaluronic acid substrate solution was added, and the mixture was incubated at 37 °C for 45 min. The reaction was terminated by adding 100 μL of stop buffer, and the mixture was further incubated at room temperature for 10 min. The absorbance was measured at 600 nm using a UV–visible spectrophotometer [78,79,80]. The hyaluronidase inhibition activity (%) was calculated using the following equation:

$$\mathrm{Hyaluronidase}\;\mathrm{inhibitory}\;\mathrm{activity}\;(\%)=\left(1-\frac{{Abs}_{\mathrm{sample}}}{{Abs}_{\mathrm{control}}}\right)\times 100$$

A standard curve was generated, and the IC_50_ value was determined accordingly.

### 4.6. Quantitative RT-PCR Analysis

Total RNA was isolated from the treated cells utilizing an RNA Extraction Kit (#74104, Qiagen, Hilden, Germany). The purity and concentration of the extracted RNA samples were evaluated using a Nanodrop 2000 spectrophotometer (Thermo Fisher Scientific, Erlangen, Germany). The isolated RNA was subsequently reverse-transcribed into complementary DNA (cDNA) using the M-MLV cDNA synthesis kit (#EZ006M, Enzynomics, Daejeon, Republic of Korea) according to the manufacturer’s protocol on a PCR instrument (T100, Bio-Rad, Hercules, CA, USA). The synthesized cDNA was then employed as a template for quantitative real-time PCR amplification (Rotor-Gene Q, Qiagen, Hilden, Germany), conducted over 40 cycles. Each PCR cycle comprises denaturation at 95 °C for 15 s, annealing at 60 °C for 30 s, and extension at 60 °C for 30 s. The primer sequences utilized in this study are detailed in Table 4. Quantitative RT-PCR (qRT-PCR) was performed using SYBR Green qPCR PreMix (#RT500M, Enzynomics, Daejeon, Republic of Korea). Relative gene expression levels were determined by the 2^−ΔΔCT^ method, with GAPDH serving as the internal control, and all experiments were conducted three times.

### 4.7. SA-β-Gal Staining Analysis

HaCaT cells were seeded at a density of 2 × 10^5^ cells into one well of a 6-well plate, while H_2_O_2_-untreated negative control cells were seeded at 1 × 10^5^ cells per well to avoid over-confluence. To induce senescence, cells were exposed to 50 μM H_2_O_2_ for 2 h, followed by two washes and subsequent culture for 3 days. NGT-M001 (1.1 μM, 25 μg/mL) and NGT-M004 (3.125 μM, 25 μg/mL) were administered 2 h before H_2_O_2_ treatment. HaCaT cells were fixed for 15 min with 2% formaldehyde (#F8775, Sigma-Aldrich, St. Louis, MO, USA) and 0.2% glutaraldehyde (#G6257, Sigma-Aldrich, St. Louis, MO, USA) in PBS. Cells were then washed and exposed overnight at 37 °C to a solution containing 1 mg/mL 5-bromo-4-chloro-3-indolyl-β-D-galactopyranoside (X-gal; #B4252, Sigma-Aldrich, St. Louis, MO, USA), 150 mM NaCl, 2 mM MgCl_2_, and 20 mM phosphate buffer, pH 6.0. Following PBS washing of the cells in X-gal staining solution, images were acquired with transmitted light microscopy (CKX53, Olympus, Tokyo, Japan) using a 4× objective, at 2560 × 1920 pixels resolution and 40% of maximum lamp intensity. The percentage of SA-β-gal-positive cells was quantified using the Fiji (ImageJ distribution, version: Fiji-Latest, downloaded from https://fiji.sc on 7 December 2025) cell counter plugin.

### 4.8. Thermostability Test

To evaluate thermostability, L-Ascorbic acid, NGT-M001, and NGT-M004 were dissolved in distilled water and incubated in a water bath (BW-10G, Jeiotech, Daejeon, Republic of Korea) at 4, 60, 70, 80, and 90 °C for a total of 2 h [81,82]. ABTS radical scavenging activity was measured.

### 4.9. Photostability Test

The photostability was performed using a light chamber with a 225 μW/cm^2^ UVB lamp source (CL-508, Uvitec, Cambridge, UK). While ISO 22643 prescribes a UVB irradiation intensity of 1.0 W/m^2^ (1000 μW/cm^2^) as the standard condition, the present study employed a reduced intensity of 225 μW/cm^2^. This adjustment was made to align the degradation rate of ascorbic acid, utilized as a reference antioxidant, with the experimental parameters. Solutions of 50 μM L-Ascorbic acid, NGT-M001 and NGT-M004 were prepared in distilled water, adjusted to pH 7.4 and placed in a 60 mm Petri dish without a cap and then exposed to radiation for 0, 5, 10, 20, 40, 80, 160 min. After radiation wash completed, the absorption of solution was measured at λ 230–400 nm using a UV-Vis spectrophotometer [83,84].

### 4.10. Cell Culture

The HaCaT cell line (human skin keratinocyte) was obtained from the AddexBio Technologies (San Diego, CA, USA). Cells were cultured in Dolbecco’s Modified Eagles Medium (DMEM; #SH30243.01, Cytiva, Marlborough, MA, USA) supplemented with 10% fetal bovine serum (FBS; #16000-044, Gibco, Grand Island, NY, USA) and 1% penicillin–streptomycin (#15140-22, Gibco, Grand Island, NY, USA). Cells were maintained in a humidified incubator at 37 °C with 5% CO_2_.

### 4.11. MTT Assay on HaCaT Cell

Cell viability was evaluated using the 3-(4,5-dimethylthiazol-2-yl)-2,5-diphenyltetrazolium bromide (MTT; #M6469, Invitrogen, Waltham, MA, USA) assay. HaCaT cells were seeded in 24-well plates at a density of 2 × 10^4^ cells per well and incubated at 37 °C in a humidified 5% CO_2_ incubator for 24 h. After incubation, the wells were washed once with PBS, and cells were treated with diluted test samples at concentrations ranging from 62.5 to 1000 µg/mL. As a negative control, some wells were filled with DMEM supplemented with 10% FBS and 1% penicillin–streptomycin. Following 24 h of treatment, the wells were washed once with PBS, and 100 µL of MTT solution (1 mg/mL in culture medium) was added to each well. The plates were incubated for 2 h at 37 °C in a 5% CO_2_ incubator. After incubation, the MTT solution was removed, and 500 µL of 100% dimethyl sulfoxide (DMSO; #472301, Sigma-Aldrich, St. Louis, MO, USA) was added to dissolve the formazan crystals. Then, 100 µL of the dissolved solution was transferred to a 96-well plate, and absorbance was measured at 570 nm using a multi-mode microplate reader (Biotek Synergy Neo2, Agilent Technologies, Winooski, VT, USA). Cell viability was calculated as the percentage ratio of the absorbance of the sample-treated wells to that of the negative control wells. CC_50_ values were determined based on cell viability measured by the MTT assay. CC_50_ was calculated by linear interpolation between the two concentrations immediately above and below 50% cell viability. Specifically, *X*_1_ and *X*_2_ represent the concentrations showing viability values above and below 50%, respectively, and *Y*_1_ and *Y*_2_ are the corresponding viability values at each concentration. The CC_50_ value was calculated using the following equation:

$${\mathrm{CC}}_{50}=X_{1}+\left(50-Y_{1}\right)\frac{\left(X_{2}-X_{1}\right)}{\left(Y_{2}-Y_{1}\right)}$$

### 4.12. Human Skin Permeability

Skin permeation was quantitatively evaluated using a Franz diffusion cell system (FDC-6TA, LOGAN Instruments, Somerset, NJ, USA) [85,86]. The apparatus consisted of a donor chamber (for sample loading), a receptor chamber (containing PBS), and a sampling port. The assay was conducted at room temperature over a 16 h period. In the donor chamber, 4 mL of each test sample (2 mg/mL, 0.2%) was loaded. The receptor chamber was filled with 10 mL of phosphate-buffered saline (PBS). Cadaver skin (female, 62 years old, back site, DermaLab™, #SDE0202; Seed Group, Seoul, Republic of Korea) was used as the membrane for the diffusion assay. The amount of permeated sample was quantitatively analyzed using a standard calibration curve prepared for each compound. The control group used L-ascorbic acid and gelatin (#G7041, Sigma-Aldrich, St. Louis, MO, USA). The skin permeation rate (%) of each test sample was calculated using the following equation:

$$\mathrm{Skin}\;\mathrm{permeability}\;(\%)=\left(\frac{C_{\mathrm{receptor}}\times 10}{C_{\mathrm{donor}}\times 4}\right)\times 100$$

### 4.13. Three-Dimensional (3D) Cell Culture

Samples were prepared by mixing various concentrations with a fixed 2% hyaluronic acid solution [87,88]. Each mixture was dispensed at 400 µL per well into a 48-well plate. Subsequently, 400 µL of 2× Minimum Essential Medium (MEM; #SH30024.01, Cytiva, Marlborough, MA, USA), supplemented with 10% FBS and 1% penicillin–streptomycin, was added to each well.

The L929 cell line (mouse fibroblast) was obtained from the Korean Cell Line Bank (KCLB; #10001, Seoul, Republic of Korea). Cells were seeded into 48-well plates at a density of 4 × 10^4^ cells per well and incubated at 37 °C in a humidified atmosphere containing 5% CO_2_ for 78 h. After incubation, 100 µL of MTT solution (1 mg/mL in culture medium) was added to each well, followed by a 2 h incubation at 37 °C in a 5% CO_2_ incubator. After removing the MTT solution, wells were washed twice with PBS and observed under an optical microscope.

### 4.14. Statistical Analysis

Statistical analyses were conducted using GraphPad Prism 8 software (San Diego, CA, USA). Data comparisons between groups were evaluated using one-way analysis of variance (ANOVA) followed by Tukey’s post hoc test for multiple comparisons. Statistical significance was defined as *p* < 0.05, with significance levels indicated as follows: * *p* < 0.05, ** *p* < 0.01, *** *p* < 0.001, and **** *p* < 0.0001. Results with *p* > 0.05 were considered not significant (ns).

## 5. Conclusions

Marine-derived bioactive compounds have been demonstrated to exert anti-aging effects through multiple mechanisms, including antioxidant activity, enhancement of the body’s intrinsic defense systems, improvement of mitochondrial function, and increased insulin sensitivity, all of which contribute to the deceleration of the aging process. Among these, marine-sourced bioactive proteins, such as recombinant mussel adhesive proteins (rMAPs), represent promising candidates that may overcome certain limitations associated with synthetic or plant-based antioxidants. These proteins hold potential as key components in the development of next-generation cosmetic and pharmaceutical formulations. Notably, the unique chemical architectures inherent to marine-origin compounds confer distinct bioactivities that differentiate them from their terrestrial analogs. For instance, mussel adhesive proteins exemplify this distinction by combining robust adhesive properties with antioxidant and anti-aging functionalities, enabling prolonged skin attachment and sustained protective effects. Specifically, recombinant MAPs NGT-M001 and NGT-M004 have demonstrated markedly enhanced antioxidant capacity—exceeding up to 27.76-fold in the ABTS assay—alongside exceptional thermal and photostability, as well as significant anti-aging efficacy. Importantly, these antioxidant effects were achieved even in the absence of DOPA residues, through synergistic contributions of tyrosine and cysteine residues, thereby underscoring the potential of marine-derived proteins as innovative functional ingredients for cosmetic and pharmaceutical applications. Continued research focusing on elucidating molecular mechanisms and conducting comprehensive efficacy evaluations is anticipated to further facilitate the commercial development of these recombinant proteins.

## Figures and Tables

**Table 1:** Evaluation of IC_50_ values in radical scavenging assay.

Sample	IC_50_ Value (μM)		μM TE/μM (TEAC) ^A^	
	ABTS	DPPH	ABTS	DPPH
Glutathione (reduced)	9.15 ± 0.05	763.23 ± 80.97	1.093	0.025
N-Acetyl Cysteine	5.19 ± 0.08	447.83 ± 27.38	1.928	0.043
Trolox	10.01 ± 0.07	19.47 ± 1.49	1	1
Ascorbic acid	14.2 ± 0.13	20.74 ± 0.16	0.701	0.938
Tocopherol	9.03 ± 0.08	14.15 ± 0.08	1.107	1.376
Resveratrol	4.3 ± 0.34	210.61 ± 5.91	2.328	0.092
Retinol	23.07 ± 2.07	2752.78 ± 495.53	0.433	0.007
rMAPs				
NGT-M001	0.38 ± 0.01	N.D ^B^	26.413	N.D
NGT-M002	1.06 ± 0.0	5.66 ± 1.12	9.436	3.437
NGT-M003	3.52 ± 0.15	12.48 ± 1.29	2.843	1.559
NGT-M004	1.1 ± 0.01	N.D	9.059	N.D
Amino acid				
L-Cysteine	12.67 ± 0.57	164.96 ± 20.56	0.789	0.118
L-Tyrosine	32.22 ± 5.55	N.D	0.31	N.D
L-DOPA	6.24 ± 0.04	54.99 ± 0.61	1.602	0.354

^A^ TEAC: Trolox equivalent antioxidant capacity, ^B^ N.D: Not detected.

**Table 2:** Kinetic evaluation of antioxidant activity based on AUC (area under the curve).

Sample	V_0_ (%/min)	AUC (%·min)	AEAC ^A^ (V_0_)	AEAC (AUC)
L-Ascorbic acid	0.24	88	1.00	1.00
N-Acetyl Cysteine	0.9	564.47	3.78	6.41
rMAPs				
NGT-M001	8.37	4953.7	35.06	56.29
NGT-M002	6.47	3795.13	27.10	43.12
NGT-M003	2.00	1293.4	8.41	14.69
NGT-M004	5.35	3552.18	22.40	40.36

^A^ L-Ascorbic acid equivalent antioxidant capacity.

**Table 3:** Skin permeability and cell cytotoxicity of MAPs.

Sample	CC_50_ (μM)	Skin Permeability (%)
L-Ascorbic acid	-	3.484 ± 0.182
Gelatin (60 kDa)	-	0.719 ± 0.006
NGT-M001	23.9 ± 2.82	1.88 ± 0.16
NGT-M004	>125	1.05 ± 0.10

**Table 4:** Strains, Plasmid and oligonucleotide primers used in this study.

Strains, Plasmid, Primers	Genotypes, Detailed Descriptions, and Sequences	Source or References
Strains		
*E. coli* DH5α	F^−^ Φ80 *lacZ*ΔM15 Δ*(lacZYA-argF)U169 deoR recA1 endA1 hsdR17(r_k_^−^, m_k_^+^) phoA supE44 thi-1 gyrA96 relA1*	Enzynomics
*E. coli* BL21 (DE3)	F^−^ *ompT hsdS*_B_(r_B_^−^ m_B_^−^) *gal dcm lon* λ(DE3), carrying the T7 RNA polymerase gene and lacIq	Novagen
Plasmids		
pET-22b(+)	T7*lac* promoter, pBR322 *ori*, Amp^r^, PelB signal sequence	Novagen
pET-NGT-M001	FP-151 gene	[34] and this study
pET-NGT-M002	*Perna viridis* foot protein-5 gene (pvFP-5)	[33] and this study
pET-NGT-M003	pvFP-5 fragment gene	This study
pET-NGT-M004	Mytilus galloprovincialis foot protein-1 (mgFP-1) gene	This study
Primer ^A^		
NGT-M003	Forward: AAAA**CATATG**TGTCGTAACGGCGGCACCTGTAAAAAACGC Reverse: AAAA**CTCGAG**TCAGTGGTGGTGGTGGTGGTGGTAGTAACCGTACGGGCAAGAGCATTTATAGTACGGG	This study
NGT-M004	Forward: GGG**CATATG**GCGAAACCGAGCTATCCG Reverse: GGG**CTCGAG**TTTGTATGTCGGCGGGTAAGACG	This study
GAPDH	Forward: GTCTCCTCTGACTTCAACAGCG Reverse: ACCACCCTGTTGCTGTAGCCAA	-
P21	Forward: AGGTGGACCTGGAGACTCTCAG Reverse: TCCTCTTGGAGAAGATCAGCCG	-

^A^ Restriction enzyme sites are bolded and 6X His tag sequences are underlined, gene names are shown in italics.

## Data Availability

The original contributions presented in this study are included in the article. Further inquiries can be directed to the corresponding authors.

## Supplementary Materials

The following supporting information can be downloaded at: https://www.mdpi.com/article/10.3390/ijms262411947/s1.

## Abbreviations

The following abbreviations are used in this manuscript:

ROS	Reactive Oxygen Species
DOPA	3,4-Dihydroxyphenylalanine
FP	Foot protein
rMAP	recombinant Mussel Adhesive Protein
CAGR	Compound Annual Growth Rate
IPTG	Isopropyl-β-D-thiogalactopyranoside
TEAC	Trolox Equivalent Antioxidant Capacity
AEAC	Ascorbic acid Equivalent Antioxidant Capacity
AUC	Area Under the Curve
ABTS	2′-Azino-bis(3-ethylbenzothiazoline-6-sulfonic acid)
DPPH	2,2-Diphenyl-1-picrylhydrazyl
EGCG	Epigallocatechin gallate
OA	Olenolic acid
UA	Ursolic acid
IDP	Intrinsically Disordered Proteins
UVB	Ultraviolet B
N.D	Not Detected
