# Supplementary material for: Development and Functional Characterization of Recombinant Mussel Adhesive Protein for Anti-Oxidative and Anti-Aging Therapeutic Applications
Source: Int J Mol Sci. 2025 Dec 11;26(24):11947. doi: 10.3390/ijms262411947 (PMC12732409; doi:10.3390/ijms262411947)
Supplement: Supplementary file 1 [file ijms-26-11947-s001.zip › ijms-3971731-supplementary.pdf]

Supplementary Materials

Development and Functional Characterization of Recombinant Mussel Adhesive Protein for Anti-Oxidative and Anti-Aging Therapeutic Applications

Suhan Wi [†], Seon-A Lim [†], Jin-Yeong Jung, Hyungmo Yang, Sun-Ae Lee, Kyounghun Choi, Ju-Ryeong Kim, Moo-Hak Lim, Yong-Hyun Kim, Jaehong Park, SeongMin Ha and Yun Heo *

Nature Gluetech Co., Ltd., 196, Gasan digital 1-ro, Geumcheon-gu, Seoul 08502, Republic of Korea; wisu@natureglue.com (W.S.); plok016@natureglue.com (S.-A.L.); ginyung92@natureglue.com (J.-Y.J.); ttkkyang91@natureglue.com (H.Y.); sun@natureglue.com (S.-A.L.); kyonghun11@natureglue.com (K.C.); joo@natureglue.com (J.-R.K.); moo@natureglue.com (M.-H.L.); yongkim22@natureglue.com (Y.-H.K.); jaehong@natureglue.com (J.P.); hsng20@natureglue.com (S.M.H.)

[*]Correspondence: yheo@natureglue.com (Y.H.)

[†]These authors contributed equally to this work.

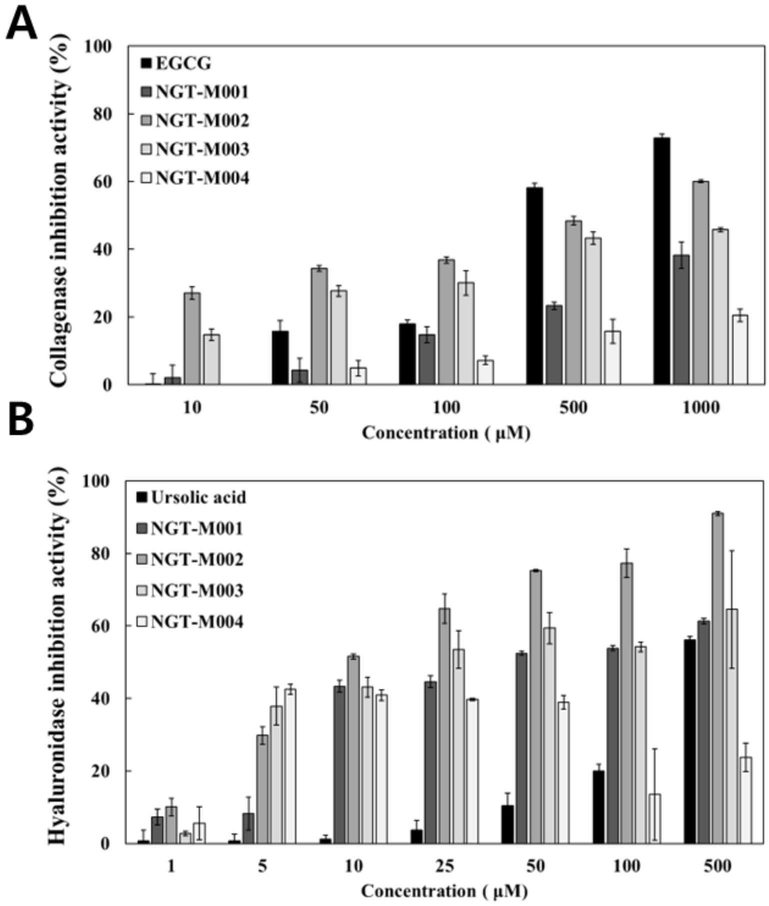

Figure S1. Evaluation of enzyme inhibitory activity according to concentration. Collagenase inhibitory activity (A) and Hyaluronidase inhibitory activity (B) of rMAPs. Data are presented as mean ± SD (n = 3).

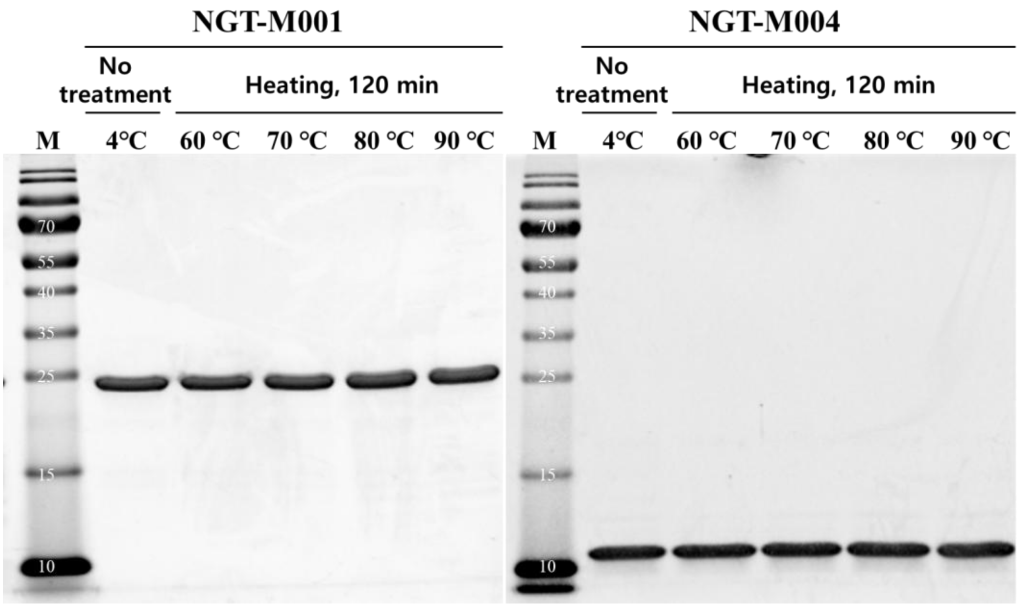

Figure S2. Protein degradation was analyzed by SDS-PAGE.

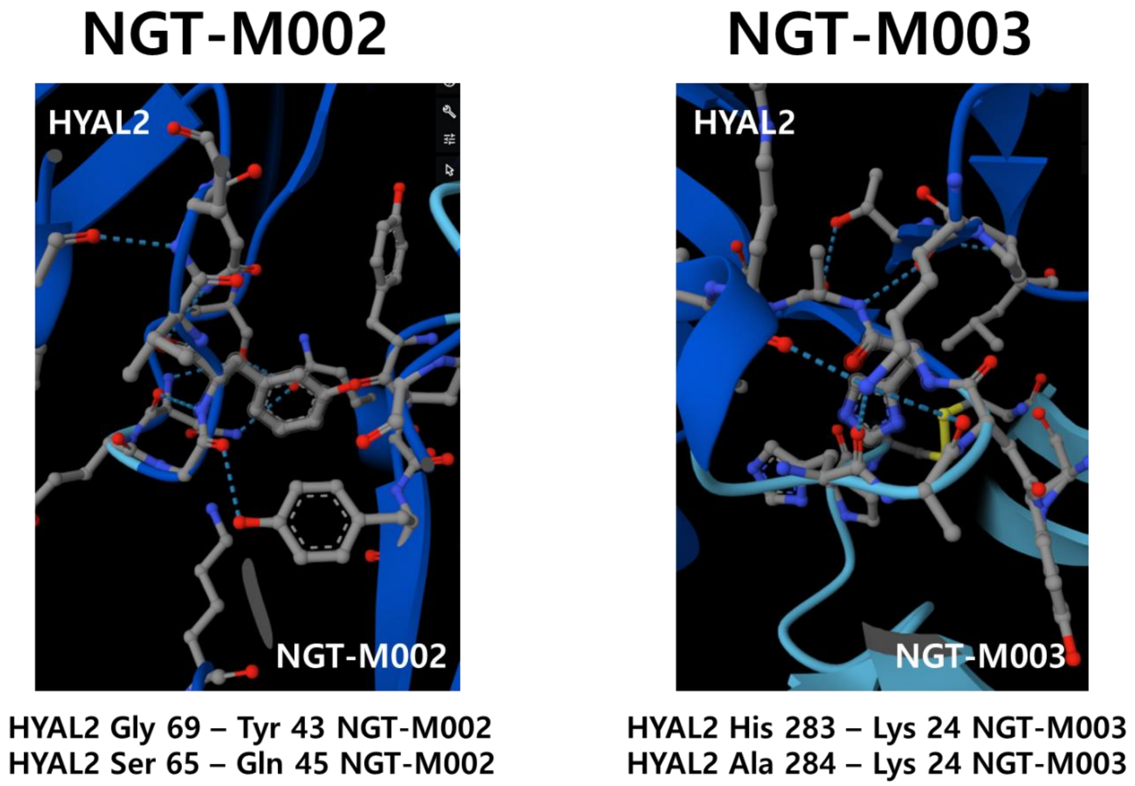

Figure S3. Protein – protein interaction was prediction by Lightdock. NGT-M002 and NGT-M003 are predicted to bind to specific sites of hyaluronidase-2 (HYAL2).
